# Liver transplantation in the critically ill: a multicenter Canadian retrospective cohort study

**DOI:** 10.1186/cc12508

**Published:** 2013-02-09

**Authors:** Constantine J Karvellas, Thomas Lescot, Peter Goldberg, Michael D Sharpe, Juan J Ronco, Eberhard L Renner, Hina Vahidy, Zafrina Poonja, Prosanto Chaudhury, Norman M Kneteman, Markus Selzner, Earl F Cook, Sean M Bagshaw

**Affiliations:** 1 Division of Critical Care Medicine, Faculty of Medicine and Dentistry, University of Alberta, 3C1.12 Walter C Mackenzie Center, 8440-112 ST NW, Edmonton, Alberta, T6G 2B7, Canada; 2 Division of Hepatology, Department of Medicine, Faculty of Medicine and Dentistry, University of Alberta, 130 University Campus NW, Edmonton, Alberta, T6G-2X8, Canada; 3 Division of Critical Care Medicine, McGill University, McGill University Health Center, 687 Pine Avenue West, Montreal, Quebec, H3A 1A1, Canada; 4 Division of Critical Care Medicine, Western University, London, Ontario, Canada; 5 Division of Critical Care Medicine, University of British Columbia, Vancouver General Hospital, 855 West 12th Avenue, Vancouver, British Columbia, V5Z 1M9, Canada; 6 Multi-Organ Transplant Program, University Health Network/University of Toronto, 585 University Avenue, Toronto, Ontario, M5G 2N2, Canada; 7 Division of Solid Organ Transplantation, McGill University Health Center, 687 Pine Avenue West, Montreal, Quebec, H3A 1A1, Canada; 8 Division of Transplantation, Department of Surgery, University of Alberta, 2D4.44 Walter C. Mackenzie Centre, Edmonton, Alberta, T6G 2B7, Canada; 9 Department of Epidemiology, Harvard School of Public Health, 677 Huntington Avenue, Boston, Massachusetts 02115, USA

## Abstract

**Introduction:**

Critically ill cirrhosis patients awaiting liver transplantation (LT) often receive prioritization for organ allocation. Identification of patients most likely to benefit is essential. The purpose of this study was to examine whether the Sequential Organ Failure Assessment (SOFA) score can predict 90-day mortality in critically ill recipients of LT and whether it can predict receipt of LT among critically ill cirrhosis listed awaiting LT.

**Methods:**

We performed a multicenter retrospective cohort study consisting of two datasets: (a) all critically-ill cirrhosis patients requiring intensive care unit (ICU) admission before LT at five transplant centers in Canada from 2000 through 2009 (one site, 1990 through 2009), and (b) critically ill cirrhosis patients receiving LT from ICU (*n *= 115) and those listed but not receiving LT before death (*n *= 106) from two centers where complete data were available.

**Results:**

In the first dataset, 198 critically ill cirrhosis patients receiving LT (mean (SD) age 53 (10) years, 66% male, median (IQR) model for end-stage liver disease (MELD) 34 (26-39)) were included. Mean (SD) SOFA scores at ICU admission, at 48 hours, and at LT were 12.5 (4), 13.0 (5), and 14.0 (4). Survival at 90 days was 84% (*n *= 166). In multivariable analysis, only older age was independently associated with reduced 90-day survival (odds ratio (OR), 1.07; 95% CI, 1.01 to 1.14; *P *= 0.013). SOFA score did not predict 90-day mortality at any time. In the second dataset, 47.9% (*n *= 106) of cirrhosis patients listed for LT died in the ICU waiting for LT. In multivariable analysis, higher SOFA at 48 hours after admission was independently associated with lower probability of receiving LT (OR, 0.89; 95% CI, 0.82 to 0.97; *P *= 0.006). When including serum lactate and SOFA at 48 hours in the final model, elevated lactate (at 48 hours) was also significantly associated with lower likelihood of receiving LT (0.32; 0.17 to 0.61; *P *= 0.001).

**Conclusions:**

SOFA appears poor at predicting 90-day survival in critically ill cirrhosis patients after LT, but higher SOFA score and elevated lactate 48 hours after ICU admission are associated with a lower probability receiving LT. Older critically ill cirrhosis patients (older than 60) receiving LT have worse 90-day survival and should be considered for LT with caution.

## Introduction

Liver transplantation (LT) for established cirrhosis is associated with good outcomes, reaching survival of greater than 80% at 1 year [1]. However, given the increasing waiting times for LT, some patients deteriorate, precipitating admission to the intensive care unit (ICU) [2]. Given the scarcity of organs and costs of prolonged ICU support, being able to discriminate those cirrhosis patients who will maximally benefit from LT after ICU admission would be useful. Therefore, objective measures to score risk of death and major morbidity based on accessible physiological and laboratory values would be useful to guide clinical decision making for liver organ allocation and transplantation among decompensated cirrhosis patients receiving support in a critical care setting [3].

Liver-specific scoring systems, such as the Child-Turcotte Pugh (CTP) score and the Model for End-stage Liver Disease (MELD), seem optimal for prognostication in slowly decompensating cirrhosis patients but may not perform as well in those with additional organ dysfunction. The CTP score, validated in cirrhosis patients undergoing surgical esophageal varix ligation/TIPS, includes synthetic hepatic markers (INR, bilirubin, albumin) and complications specific to cirrhosis (ascites and encephalopathy) but does not take into account cardiac, pulmonary, and/or kidney dysfunction (that is, non-liver organ dysfunction) [3-6]. MELD is currently used for organ allocation in North America and has been validated for 3-month survival in cirrhosis patients (all etiologies) without transplant, but its ability to prognosticate outcome with transplant in critically ill cirrhosis patients has not been rigorously evaluated [7,8].

Illness severity scores such as the Acute Physiology and Chronic Health Evaluation (APACHE) II score (see operational definitions) or the Sequential Organ Failure Assessment (SOFA) could potentially offer superior prognostication in the setting of extrahepatic organ dysfunction. APACHE II correlates with hospital outcome in a mixed ICU population and in nontransplanted cirrhosis patients; however, it has been validated for use only at the time of ICU admission [9,10]. SOFA is a validated ICU-specific organ-dysfunction score, developed in a heterogeneous critically ill septic population, and correlates with outcome [10,11]. The SOFA score, calculated as a composite of gradations in severity of organ dysfunction across six organ systems (neurologic, respiratory, cardiovascular, renal, hematologic, and hepatic), can be calculated daily to assess evolution of organ dysfunction [11]. Prior studies showed that SOFA scores above 11 correspond to hospital mortality exceeding 80% in a heterogeneous ICU population [12]. To date; however, the clinical utility of the SOFA score to predict outcomes in critically ill cirrhosis patients has been evaluated only in patients that did not receive LT [13-16].

We hypothesized that increased severity of organ dysfunction, as defined by the SOFA score, would adversely affect 90-day survival and reduce the proportion of listed eligible critically ill cirrhosis patients receiving LT. Accordingly, our objectives were:

1. To determine whether critically ill cirrhosis patients with a high burden of organ dysfunction, as defined by the SOFA score, measured at the time of ICU admission, at 48 hours after admission, and on the day of LT, have a higher 90-day mortality compared with those with less-severe organ dysfunction.

2. To determine whether critically ill cirrhosis patients with a high burden of organ dysfunction, as defined by the SOFA score, measured at the time of ICU admission and at 48 hours after admission, who are eligible for LT, are less likely to receive LT compared with those with less-severe organ dysfunction.

## Materials and methods

The reporting of this study follows the STROBE statement for observational studies [17]. The Health Research Ethics Boards at each participating institution approved this study before commencement. The requirement for individual informed consent was waived.

### Design and setting

For our first objective, we performed a retrospective cohort study of all critically ill cirrhosis patients transplanted (*n *= 198) at five major Canadian liver transplant centers (University of Alberta, Edmonton, Alberta; University of British Columbia, Vancouver, British Columbia; McGill University, Montreal, Quebec; University of Toronto, Toronto, Ontario; and Western University, London, Ontario) between January 2000 and December 2009. Data were collected from the University of Alberta for all cirrhosis patients given transplants between January 1990 and December 2009.

For our second objective, we performed a retrospective cohort study from two LT centers (University of Alberta, Edmonton, from January 1990 through December 2009; McGill University, Montreal, from January 2000 through December 2009) where complete data were available on all critically ill cirrhosis patients who received an LT while in the ICU (*n *= 115) and from those eligible cirrhosis patients listed for LT but who did not receive an LT and died while in the ICU (*n *= 106). Data from the other three centers on eligible critically ill cirrhosis patients listed but who did not receive LT while in the ICU were unavailable.

### Participants

Inclusion criteria were as follows: (a) prior diagnosis of cirrhosis AND listed for LT; (b) age ≥18 years; and (c) admission to an ICU with critical illness/organ failures.

Exclusion criteria were as follows: (a) primary diagnosis of acute (fulminant) liver failure; and (b) liver transplantation from the ward or home; (c) patients receiving retransplant; and (d) multiorgan transplant (for example, liver and kidney).

### Operational definitions

The Sequential Organ Failure Assessment (SOFA) score is an organ-failure scoring system comprising six organ-system domains (neurologic, respiratory, cardiovascular, renal, hematologic, and hepatic) [11]. Each organ system earns a score out of 4 (range, 0 to 4, with 4 being the worst) and with a maximum score out of 24. The Acute Physiology and Chronic Health Evaluation (APACHE) II Score is a illness-severity classification system based on initial values of 12 routine physiologic measurements, age, and previous health status to provide a general measure of severity of disease. An increasing score (range, 0 to 71) correlates with increasing risk of hospital death [10]. The Charlson co-morbidity index (CCI) is a validated summary measure of premorbid chronic disease that correlates with 1-year mortality. The CCI comprises 22 different chronic conditions. Each condition is assigned a score (range, 1, 2, 3, or 6, depending on the severity and the risk of dying associated with this condition) and added to provide a composite score [18]. We used a modified version of this by excluding liver disease. The Donor Risk Index (DRI) is a composite score of donor variables derived from 9,882 deceased LT donors (April 1, 2002 to December 31, 2003) from the Scientific Registry of Transplant Recipients (SRTR) database [19]. Given limited donor information from chart review, we used a modified composite score of variables that were available and based on the DRI (donor age >60, partial/split-liver graft, cold ischemia time >8 hours, stroke/cerebrovascular accident as principal donor cause of death (either ischemic or hemorrhagic)).

### Variables

Our primary exposure of interest was severity of organ dysfunction, as defined by the SOFA score, sequentially assessed after ICU admission [11]. For the LT cohort (objective 1; *n *= 198), the SOFA score was assessed at ICU admission, at 48 hours after admission, and on the day of LT. For the eligible but non-LT cohort (objective 2; *n *= 106), the SOFA score was assessed at ICU admission and at 48 hours after admission.

### Data sources and collection

Data were extracted from patient medical records and existing regional liver-transplant databases. Data fields abstracted included the etiology and complications of cirrhosis, preoperative (admission, 48 hours, day of transplant, if applicable) hematologic, biochemical and physiological/organ-dysfunction data (requirement for vasopressors, mechanical ventilation, renal replacement therapy (RRT)), donor information, and outcomes after LT (see Table 1). Data were collected on medical comorbidities (modified CCI) when available. Patient outcomes collected included surgical complications of transplant, duration of mechanical ventilation, duration of ICU/hospital stay, need for postoperative RRT, and mortality.

**Table 1:** Baseline, donor, and outcome characteristics for 198 transplanted critically ill cirrhosis patients (first objective, five sites) and 106 nontransplanted critically ill cirrhosis patients (second objective, two sites)

	First objective (*n *= 198)	Second objective (*n *= 106)
Age (years)	53 (10)	54 (9.5)

Female	67 (34%)	31 (29%)

Etiology		

Hepatitis C	62 (31%)	30 (29%)
Hepatitis B	17 (9%)	7 (7%)
Alcohol	30 (15%)	24 (23%)
PSC/PBC	30 (15%)	9 (9%)
NASH/Cryptogenic	17 (9%)	18 (17%)

Comorbidities/Cirrhotic complications		

Charlson Score	1 (1)	0.7 (1)
Ascites	100 (96%)	61 (71%)
Variceal bleeding	53 (56%)	54 (64%)
Hepatic encephalopathy	107 (94%)	67 (79%)
Hepatorenal syndrome	84 (63%)	71 (69%)
Spontaneous bacterial peritonitis	34 (41%)	37 (44%)

Hematology		

Hemoglobin (g/L)	85 (23)	84 (22)
White blood count (×10^9^/L)	8.9 (5.2-14.5)	9.7 (6.5-15.4)
Platelet count (×10^9^/L)	64 (43-95)	70 (40-118)

Biochemistry		

INR	2.1 (1.7-2.8)	2.2 (1.8-3.3)
ALT (U/L)	46 (25-82)	53 (27-128)
Bilirubin (μ*M*)	273 (95-575)	239 (95-469)
Sodium (m*M*)	137 (130-143)	136 (130-143)
Lactate (m*M*)	2.8 (1.6-4.6)	3.6 (2.4-7.8)
pH	7.39 (7.32-7.46)	7.36 (7.25-7.44)
Creatinine (μ*M*)	197 (109-308)	207 (122-301)

Physiology		

Mean arterial pressure (mm Hg)	67 (60-83)	52 (10)
Glasgow Coma Scale score (admission)	10 (5)	9 (5)
PO_2_/FiO_2 _ratio (mm Hg, admission)	227 (106)	195 (112)

Organ support		

Vasopressors (admission)	84/186 (45%)	54/85 (64%)
Vasopressors (any day)	95/108 (88%)	58/74 (78%)
Mechanical ventilation (admission)	76/134 (57%)	50/86 (58%)
MV (any day)	100/114 (88%)	61/74 (82%)
RRT (admission)	49/187 (26%)	27/87 (31%)
RRT (any day)	78/139 (56%)	42/76 (55%)

Aggregate scores		

Child Turcotte Pugh (listing)	12.4 (1.6)	
MELD (listing)	24 (16-36)	23 (15-35)
MELD (admit)	34 (26-39)	36 (27-40)
MELD (transplant)	34 (27-40)	
SOFA (admit)	12.5 (4)	14 (4)
SOFA (48 hours)	13 (5)	17 (4)
SOFA (transplant)	14 (4)	

Mortality		

90 days	32 (16%)	
1 year	52 (26%)	
3 years	63 (38%)	

Donor characteristics		

Donor age >60	35 (18%)	
Partial graft (split)	2 (1%)	
Donor cerebrovascular accident	105 (54%)	
Cold ischemia time >8 hours	102 (56%)	
Donor score (of 4)	1 (1-2)	

Charlson score was modified to exclude liver disease.

### Statistical analysis

#### First objective: predictors of 90-day mortality after liver transplant (five sites)

For our first objective, our primary outcome was 90-day mortality among critically ill cirrhosis patients receiving LT. The rationale for ascertainment of mortality at 90 days was largely to reflect the early perioperative contributing factors (severity of illness, donor characteristics) and to avoid later confounding due to recurrent diseases, such as recurrent hepatitis C virus (HCV) or graft loss due to noncompliance with immunosuppression. The main covariates examined included age, sex, liver-disease etiology, time of listing to and time from ICU admission to LT, decade of transplant, and graft/donor risk.

In the event of missing values, data were not replaced or estimated. After normality testing of descriptive statistics, normally distributed variables were reported as means with standard deviations (SDs) and compared with the Student *t *test. Nonnormally distributed continuous data were reported as medians with interquartile ranges (IQRs) and compared with the Wilcoxon rank-sum test. Categoric events were compared by using the χ^2 ^test or the Fisher Exact test where appropriate (*n *< 5 events). A *P *value of <0.05 was be considered statistically significant for all comparisons. Analysis was performed by using IBM SPSS Version 19 (IBM, New York, USA).

A logistic regression analysis was performed on the cohort of 198 subjects to determine whether the probability of 90-day mortality was affected by SOFA in three separate models (at admission, at 48 hours, and at day of LT). Covariates included in the model were based on univariate unadjusted odds ratios (*P *< 0.1) and predefined covariates; age, sex (female), etiology of liver disease, decade of LT, and transplant site. Etiology was collapsed into hepatitis C (HCV) versus other, because of the less-favorable outcomes associated with HCV [20]. To account for changes in ICU/transplant practice, time (decade) was collapsed into a binary variable 1990 through 1999 versus 2000 through 2009, as one site (University of Alberta) had data from 20 years. Covariates in each model were assessed for collinearity. The individual parameters included in SOFA were not included in derivation of these models. Multivariate associations are reported as odds ratios (ORs) with 95% confidence limits. All presented models met criteria for fit were assessed by the Hosmer-Lemeshow goodness-of-fit test (*P *> 0.3 for all).

#### Second objective: predictors of transplant in 221 critically ill cirrhosis patients (two sites)

For our second objective, physiological data, biochemical data, and SOFA were compared (admission, 48 hours) between all cirrhosis patients transplanted from ICU and those cirrhosis patients listed for LT but who died in the ICU awaiting an organ. The primary outcome was receipt of transplant. Univariable analysis was performed as described as for the first aim.

We subsequently performed a multivariable logistic regression analysis exploring predictors of receipt of LT. Covariates and multivariate associations were reported as described for the first aim. From univariate logistic regression (*P *value of <0.10), lactate was included with predefined covariates. Lactate (admission at 48 hours) was transformed into its natural logarithm to meet the assumption of a normal distribution.

## Results

### First objective: predictors of mortality in transplanted critically ill cirrhosis patients

#### Participants and descriptive data

In total, 198 critically ill cirrhosis patients (mean (SD) age, 53 (10) years, 66% male) received transplants during the study period. Baseline characteristics are shown in Table 1. The most common etiologies were hepatitis C (31%) and alcohol abuse (15%). LT occurred a median (IQR) of 29 (5 to 101) days from listing and 5 (3 to 10) days from ICU admission. While in the ICU, 88% received vasopressors, 56% received RRT, and 87% were mechanically ventilated before LT. The median (IQR) MELD score was 34 (26 to 39) on ICU admission and 34 (27 to 40) on the day of LT, respectively. SOFA scores (mean (SD)) were 12.5 (4), 13 (5), and 14 (4) on ICU admission, at 48 hours, and on the day of LT, respectively.

Overall, 166 (84%) of all 198 transplanted critically ill cirrhosis patients were alive at 90 days, 145 (74%) of 197 at 1 year, and 105 (62.5%) of 163 at 3 years. Sixteen (8%) patients were given repeated transplants. Donor characteristics are also shown in Table 1. Thirty-five (18%) of 193 patients received a graft from a donor aged >60 years, 1% received a split graft, 54% of donors died of a cerebrovascular accident (ischemic or hemorrhagic), and 56% of donor organs were transplanted with a cold-ischemia time exceeding 8 hours.

#### Univariable outcome data: 198 liver transplant recipients

Results of univariable analysis are shown in Table 2. Comparing patients who were alive (*n *= 166, 84%) with those who were dead (*n *= 32, 16%) at 90 days, no statistically significant differences in MELD were found on admission or day of LT (*P *> 0.6 for both). No statistically significant differences were seen between SOFA on admission or day of LT (*P *> 0.17 for both). Patients alive at 90 days were significantly younger (51 versus 56 years; *P *= 0.007). Patients older than 60 years had significantly higher 90-day mortality (27% versus 13%; *P *= 0.04), and a trend toward increased 1-year mortality (37% versus 23%; *P *= 0.09). No significant differences in donor characteristics were noted comparing LT recipients alive at 90 days with nonsurvivors. The primary cause of death in patients deceased <90 days was sepsis/multiorgan failure (*n *= 7, 54% where data were available).

**Table 2:** Univariable analysis of admission and pretransplant factors for survivors versus nonsurvivors at 90 days in 198 transplanted critically ill cirrhosis patients (five sites)

	Alive at 90 days (*n *= 166)	Dead at 90 days (*n *= 32)	*P *value
Age	51 (11)	56 (8)	0.007

Age >60	30 (18%)	11 (34%)	0.037

Female	58/165 (35%)	9/32 (28%)	0.4

HCV	54/166 (33%)	8/32 (25%)	0.4

Biochemistry (admission)			

Hemoglobin (g/L)	85 (24)	86 (21)	0.9
WBC (×10^9^/L)	9.2 (5.2-14.8)	8.0 (5.5-11.8)	0.4
Platelets (×10^9^/L)	62 (41-94)	71 (49-109)	0.14
INR	2.1 (1.7-2.7)	2.4 (1.8-3.0)	0.3
Bilirubin (μ*M*)	272 (101-589)	269 (74-540)	0.4
Lactate (m*M*)	2.8 (1.7-4.6)	1.5 (1.1-4.6)	0.23
Sodium (m*M*)	136 (131-142)	136 (131-141)	0.7
Creatinine (μ*M*)	195 (97-303)	225 (141-336)	0.17

			

Physiology (admission)			

GCS	10 (4.5)	11 (4.5)	0.6
PO_2_/FiO_2 _ratio (mm Hg)	229 (102)	217 (132)	0.5
Mechanical ventilation	68/121 (56%)	8/13 (62%)	0.7
Vasoactive drugs	75/157 (48%)	9/29 (31%)	0.10
Renal replacement therapy	43/158 (27%)	6/29 (21%)	0.5

Child-Turcotte-Pugh score			

Listing	12.5 (1.4)	12.9 (1.4)	0.5

MELD			

Listing	24 (16-36)	27 (17-27)	0.85
Admission	34 (26-39)	34 (25-40)	0.6
Day of transplant	35 (26-40)	34 (28-40)	0.9

SOFA			

Admission	13 (4)	12 (5)	0.11
48 hours	14 (5)	11 (4)	0.023
Day of transplant	14 (4)	11 (4)	0.15

ICU Length of stay			

Before transplant	5 (3-10)	6 (2-14)	0.6
After transplant	9 (5-20)	8 (2-14)	0.05

Donor score (out of 4)	1 (1-2)	1 (1-2)	0.6

Partial graft	2/160 (1%)	0/30 (0)	0.5
Cold ischemia time >8 hours	88/157 (56%)	14/26 (54%)	0.8
Donor cerebrovascular accident	85/163 (52%)	20/30 (67%)	0.14
Donor age >60	29/162 (18%)	6/31 (19%)	0.8

#### Multivariable analysis: predictors of mortality

Three separate multivariable logistic regression models were built to assess the impact of the exposure SOFA on admission (model 1), SOFA 48 hours after ICU admission (model 2), and SOFA on the day of LT (model 3) and survival (Table 3). After covariate adjustment, SOFA was not significantly predictive of 90-day mortality when assessed at ICU admission, at 48 hours, or on the day of LT (*P *> 0.2 for all). In all three models, age (per incremental year) was independently associated with higher 90-day mortality in all models (*P *< 0.03 for all models) after controlling for SOFA, sex, etiology, decade of transplant, and transplant center.

**Table 3:** Multivariable analysis: predictors of 90-day mortality in 198 critically ill cirrhosis patients who underwent liver transplantation

Covariate	Unadjusted	Model 1 (*n *= 180)	Model 2 (*n *= 110)	Model 3 (*n *= 140)
Age	1.06 (1.01-1.11)^a^	1.07 (1.01-1.14)^b^	1.12 (1.02-1.22)^c^	1.07 (1.01-1.14)^d^

Gender (female)	0.72 (0.31-1.66)	0.72 (0.29-2.39)	0.24 (0.05-1.181)	0.60 (0.19-1.82)

Etiology (HCV versus non-HCV)	0.69 (0.29-1.64)	0.86 (0.30-2.40)	0.39 (0.100-1.181)	1.07 (0.37-3.07)

				

SOFA (admission)	0.93 (0.85-1.03)	1.07 (0.94-1.22)		

SOFA (48 hours)	0.88 (0.79-0.99)		1.04 (0.87-1.23)	

SOFA (day of transplant)	0.94 (0.85-1.04)			1.04 (0.90-1.19)

				

χ^2 ^(degrees of freedom)		31.00 (9)	35.73 (8)	23.81 (8)

All three multivariable models were adjusted for site (five) and decade of transplant (2000 through 2009 versus 1990 through 1990). Hosmer-Lemeshow Goodness of Fit performed well (*P *> 0.15) for all models. Model one included age, gender (female), etiology (HCV versus non-HCV) and SOFA on admission (*n *= 180/198 patients; 18 patients had missing data). Model two had similar covariates but incorporated SOFA at 48 hours after admission (*n *= 110; 88 patients had missing data). Model three had similar covariates but incorporated SOFA on the day of liver transplant (*n *= 140; 58 patients had missing data). Significant *P *values were for age (unadjusted (*P *= 0.013),^a ^Model 1 (*P *= 0.015),^b ^Model 2 (*P *= 0.014), and ^d ^Model 3 (*P *= 0.027).

### Second objective: predictors of transplant in 221 critically ill cirrhosis patients (two sites)

#### Participants and descriptive data

Baseline data of 106 nontransplanted cirrhosis patients are shown in Table 1. The most common etiologies were hepatitis C (29%) and alcohol abuse (23%). In total, 78% required vasopressors, 55% received RRT, and 82% were mechanically ventilated while in the ICU. The median (IQR) MELD score was 36 (27 to 40) on ICU admission. Mean (SD) SOFA scores were 14 (4) and 17 (4) on ICU admission and at 48 hours, respectively.

#### Univariable analysis: Liver transplant in 221 critically ill cirrhotics (two sites)

**Figure 1:**
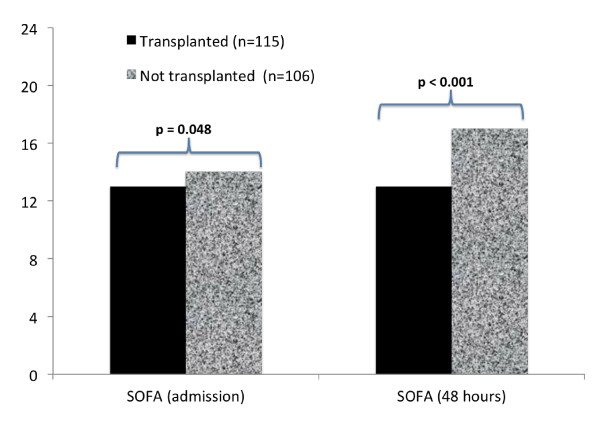
**Comparison of SOFA scores on admission and 48 hours after admission between 115 patients who received liver transplant and 106 patients who died in ICU while awaiting transplant (two sites)**.

Patients who died waiting for LT had higher MELD scores on admission (36 versus 33, *P *= 0.035; see Additional file 1). Furthermore, SOFA on admission (14 versus 13; *P *= 0.048) and at 48 hours after ICU admission (17 versus 13; *P *< 0.001) were significantly higher in patients who went on not to receive LT (See Figure 1). On ICU admission, patients who died waiting for LT had worse coagulopathy (INR 2.2 versus 2.0; *P *= 0.009), acidemia (pH 7.36 versus 7.38; *P *= 0.019) and had higher serum lactate levels (3.6 versus 2.6; *P *= 0.003). On admission, non-LT patients were more likely to be hypotensive (MAP, 54 versus 66; *P *< 0.001) and to require vasopressor support (64% versus 47%; *P *= 0.02).

#### iii) Multivariable analysis: predictors of transplant (two sites)

After covariate-adjustment, SOFA on admission was not significantly associated with receipt of LT (OR, 0.96; 95% CI, 0.89 to 1.04; *see *Additional file 2). In multivariable analysis of SOFA at 48 hours after admission, after adjustment for patient age and other covariates except lactate, higher SOFA at 48 hours was associated with a lower likelihood of receiving LT (see Table 4: OR, 0.89; 95% CI, 0.82 to 0.97; *P *= 0.006). However when lactate at 48 hours is included in the model with other covariates, 48-hour lactate appears to have a stronger association with likelihood of transplant (OR, 0.32; 95% CI, 0.17 to 0.61; *P *= 0.001) compared with SOFA (*P *= 0.19).

**Table 4:** Multivariable analysis: predictors of receipt of liver transplant in 221 critically ill cirrhosis patients on transplant list in ICU

Covariate	Unadjusted (*n *= 221)	Model 1 (*n *= 126)	Model 2 (*n *= 174)	Model 3 (*n *= 125)
Age	0.99 (0.96-1.01)	**0.95 (0.91-0.99)^a^**	0.98 (0.94-1.01)	**0.95 (0.91-0.99) ^a ^**

Gender (female)	1.06 (0.59-1.89)	1.51 (0.64-3.57)	1.02 (0.51-2.05)	1.58 (0.66-3.80)

Etiology (HCV versus non-HCV)	1.28 (0.72-2.28)	1.78 (0.76-4.14)	1.63 (0.81-3.28)	1.72 (0.73-4.03)

				

SOFA (admission)^b^	**0.94 (0.88-0.99)^a^**			

Lactate (admission, natural log)^c^	**0.52 (0.34-0.79)^a^**			

				

SOFA (48 hours)	**0.88 (0.82-0.94)^a^**		**0.89 (0.82-0.97)^a^**	0.92 (0.82-1.04)

48-hour lactate (natural log)	**0.30 (0.17-0.53)^a^**	**0.29 (0.15-0.53)^a^**		**0.32 (0.17-0.61) ^a ^**

				

χ^2 ^(degrees of freedom)		31.03 (6)	23.75 (6)	33.23 (7)

All multivariable models were adjusted for site (two sites) and decade of transplant (2000 through 2009 versus 1990 through1990). Lactate (on admission and at 48 hours) was converted to a natural logarithm (normal distribution). Hosmer-Lemeshow goodness-of-fit performed well (*P *> 0.3) for all models. All models included age, gender (female), etiology (HCV versus non-HCV). Model 1 (*n *= 126, 95 missing) included lactate (natural log) at 48 hours after admission. Model 2 (*n *= 174, 37 missing) included SOFA score at 48 hours. Model 3 (*n *= 125, 96 missing) included SOFA score and lactate (natural log) at 48 hours. Significant results (*P *values) included Age: Model 1 (*P *= 0.036), Model 3 (*P *= 0.043); SOFA (at 48 hours): Unadjusted (*P *< 0.001), Model 2 (*P *= 0.006); Lactate 48 hours after admission (natural logarithm): Unadjusted (*P *< 0.001), Model 1 (*P *< 0.001), Model 3 (*P *= 0.001). See Additional file 2 for full derivation of all models and *P *values.

## Discussion

We performed a retrospective cohort study from the five largest liver-transplant centers in Canada to evaluate the impact of critical illness and organ dysfunction on short-term mortality after LT and on organ allocation among critically ill cirrhosis patients listed for LT and admitted to the ICU.

### Key results

We found that among critically ill cirrhosis patients who received LT, older age independently portends a significantly higher risk for post-LT 90-day mortality. We also showed that SOFA score on ICU admission, at 48 hours, and on the day of LT is not predictive of 90-day mortality. When comparing those patients who received LT from the ICU with those patients who died in the ICU awaiting LT, we found that SOFA at 48 hours after ICU admission is independently associated with a lower likelihood of receiving LT. We also found that high lactate (on admission and at 48 hours) and patient age are independently associated with lower likelihood for LT.

### Comparison with previous studies

This study extends on data from previous studies as it is a multicenter study across an organ-sharing network (Canada) and specifically focused on critically ill cirrhosis patients who were listed or received LT while admitted to an ICU. In a Swiss observational study of 144 consecutive LT recipients, Oberkofler and colleagues [21] showed that a MELD >23 was predictive of increased postoperative length of ICU stay but not lower short-term survival [21]. This study, however, was limited because of being representative of a single-center study and including a heterogeneous cohort of ICU and non-ICU patients receiving LT. Given that non-ICU patients were included, only MELD (not SOFA) could be evaluated. Nonetheless, this study showed no association between MELD and post-LT outcome in the critically ill. Previously, Cholangitas and colleagues [22] showed that, in nontransplanted cirrhosis patients from a single center, prognostic scores had increased sensitivity at 48 hours rather than on admission and that SOFA outperformed MELD and APACHE II (on admission). However, this study did not directly evaluate outcomes of critically ill cirrhosis patients receiving LT from the ICU or predictors of receiving an LT. In another single-center Taiwanese study, Wong *et al. *[23] found that although the pre-LT SOFA score predicted unadjusted 3-month and 1-year survival, not all patients were critically ill and admitted to the ICU before LT, and no covariate adjustment was performed [23]. Although SOFA did not appear to be independently associated with 90-day survival in critically ill transplanted cirrhosis patients, our data did show that a higher SOFA score 48 hours after ICU admission was independently associated with a lower likelihood of receiving LT. In contrast with a single-center study from Umgelter and colleagues [24] that reported overall 90-day mortality rates of almost 40% for transplanted critically ill cirrhosis patients (*n *= 23), in our cohort, 90-day mortality was lower (16%).

### Study limitations

Our study has several limitations that warrant consideration. First, although our study is multicentric and represents five major liver-transplant centers in Canada (data not available from two other centers), it is relatively small, and a retrospective analysis of prospectively collected data is thereby potentially predisposed to selection bias and residual confounding. Second, individual centers vary in volume and make independent decisions about listing for LT; hence variations may occur in listing/de-listing practices across sites. However, the evaluation of health outcomes, such as organ allocation in critically ill cirrhosis patients and their associated LT outcomes to date has not been evaluated in a prospective manner. Furthermore, all transplant centers that provided data for this study participate in the Canadian Liver transplant organ sharing network, in which decisions are often made to transfer organs from different regions across the country for critically ill cirrhosis patients requiring LT [25]. Accordingly, replicating observational studies of this nature has value to assess for consistency and generalizability. Third, although we attempted to collect data on the donor risk index [19], our data were not complete for all patients, and we used a modified donor-risk score. Furthermore, we were unable to obtain reliable data on surgical complications and intraoperative parameters from all sites.

Finally, we acknowledge that age, as an independent predictor of increased 90-day mortality, could potentially be confounded by virtue of being a surrogate of increased comorbidity [26]. However, as opposed to other critically ill populations, LT candidates are often excluded for having significant cardiopulmonary morbidity or coexisting malignancy. Low Charlson comorbidity scores in this cohort reflected this.

In this study, we found that SOFA was not independently associated with short-term mortality after LT. Because of the complexities of decision making in LT and the inherent selection bias (that is, selected centers may inherently decline sicker patients), it remains to be determined whether SOFA is the best organ-failure score with which to prognosticate in this cohort [13,22]. Given the limitation of retrospective studies, future research should be focused on well-designed prospective studies looking at reliable collection of multiple confounding variables. With adequate patient numbers for derivation and validation of a prediction rule, a novel dynamic scoring system that includes other factors (for example, age, lactate) that are not incorporated into the SOFA score could be derived to reflect accurately characteristics that may not be shared between critically ill patients listed for LT and other critically ill populations. Important outcomes, besides mortality, also include the impact of critical illness on health-service allocation issues, such as length of time on mechanical ventilation/renal replacement therapy, rehospitalization, and persistent nonliver organ dysfunction (neurocognitive, respiratory, nutritional issues). Organ dysfunction has been previously shown to affect cost significantly in critically ill nontransplanted cirrhosis patients, and it would be useful to look at the impact on transplant recipients [27].

## Conclusions

SOFA does not appear to predict outcome after LT in this cohort. However, higher SOFA scores at 48 hours after admission, along with older age and serum lactate, are associated with a lower probability of receiving LT. Older critically ill cirrhosis patients (older than 60) undergoing LT have significantly worse 90-day post-LT mortality and should be considered for transplant with caution.

## Key messages

• SOFA does not appear to predict outcome after LT in critically ill cirrhosis patients

• Older age is significantly associated with worse early outcomes after LT in critically ill cirrhosis patients

• Higher SOFA scores at 48 hours after ICU admission are associated with a lower probability of receiving LT in critically ill cirrhosis patients listed for LT.

• Higher serum lactate at 48 hours after ICU admission is associated with a lower probability of receiving LT in critically ill cirrhosis patients listed for LT.

## Abbreviations

AoCLF: Acute-on-chronic liver failure; APACHE: Acute Physiology and Chronic Health Evaluation; CCI: Charlson Comorbidity Index; HBV: hepatitis B; HCV: hepatitis C; ICU: intensive care unit; LT: liver transplant; MELD: Model for End-stage Liver Disease; MV: mechanical ventilation; PNF: primary nonfunction; RRT: renal replacement therapy; SOFA: Sequential Organ Failure Assessment.

## Competing interests

The authors declare that they have no competing interests.

## Authors' contributions

CJK wrote and extensively revised the manuscript, compiled the final database, and performed statistical and data analyses. TL, PG, MDS, JJR, ELR, NMK, PC, and MS provided content expertise and assisted in compiling the database, manuscript editing, and revision. HV and ZP assisted in compiling the database and manuscript revision. EFC was a statistical advisor for the study and assisted with significant manuscript revision. SMB provided content expertise, significant guidance on analysis and interpretation of data, and assisted extensively with manuscript revision. All authors read and approved the final manuscript for publication.

## Supplementary Material

Additional file 1 **Univariable analysis comparing 115 transplanted cirrhosis patients with106 cirrhosis patients listed but who died while waiting for transplant (two sites)**. Demographic, biochemical, and physiological comparisons between transplanted and nontransplanted cirrhosis patients (unadjusted). Click here for file

Additional file 2 Multivariable analysis: Predictors of receipt of liver transplant in 221 critically ill cirrhosis patients on the transplant list in the ICU.Description: Multivariable (adjusted) predictors of liver transplantation (2 sites). Click here for file
