# Supplementary material for: Liver transplantation in the critically ill: a multicenter Canadian retrospective cohort study
Source: Crit Care. 2013 Feb 9;17(1):R28. doi: 10.1186/cc12508 (PMC4056692; doi:10.1186/cc12508)
Supplement: Additional file 1 — Univariable analysis comparing 115 transplanted cirrhosis patients with106 cirrhosis patients listed but who died while waiting for transplant (two sites). Demographic, biochemical, and physiological comparisons between transplanted and nontransplanted cirrhosis patients (unadjusted). [file cc12508-S1.DOCX]

|  | Transplanted  (n=115) | Listed Not transplanted  (n=106) | P-value |
| --- | --- | --- | --- |
| Age | 51 (10) | 52 (10) | 0.3 |
| Age> 60 | 22/115 (19%) | 24/106 (23%) | 0.5 |
| Female | 35/115 (30%) | 31/106 (29%) | 0.9 |
| HCV | 39/115 (34%) | 30/106 (29%) | 0.4 |
| Biochemistry (admission) |  |  |  |
| Hemoglobin (g/L) | 84 (28) | 84(22) | 0.6 |
| White Blood Count (x 10^9^/L) | 9.2 (5.4-13.5) | 9.7 (6.5-15.4) | 0.09 |
| Platelets (x 10^9^/L) | 64 (43-97) | 70 (40-118) | 0.6 |
| INR | 2.0 (1.6-2.7) | 2.2 (1.8-3.3) | 0.009 |
| Bilirubin (**μ**mol/L) | 236 (73-587) | 239 (95-469) | 0.9 |
| Lactate (mmol/L) | 2.6 (1.6-4.6) | 3.6 (2.4-7.8) | 0.003 |
| Sodium (mmol/L) | 136 (8) | 136 (9) | 0.9 |
| Creatinine (**μ**mol/L) | 196 (85-306) | 207 (122-301) | 0.5 |
| pH | 7.38 (7.32-7.46) | 7.36 (7.25-7.44) | 0.019 |
| Physiology (admission) |  |  |  |
| Mean arterial pressure (mm Hg) | 66 (59-76) | 54 (46-60) | <0.001 |
| GCS | 10 (5) | 9 (5) | 0.15 |
| PO_2_/FiO_2_ ratio (mm Hg) | 216 (99) | 195 (112) | 0.07 |
| Mechanical ventilation | 44/74 (60%) | 50/86 (58%) | 0.9 |
| Vasoactive drugs | 52/110 (47%) | 54/85 (64%) | 0.024 |
| Renal replacement therapy | 29/111 (26%) | 27/87 (31%) | 0.5 |
| MELD |  |  |  |
| Admission | 33(24-39) | 36 (27-40) | 0.035 |
| SOFA |  |  |  |
| Admission | 13 (5) | 14 (4) | 0.048 |
| 48 hours | 13(5) | 17 (4) | <0.001 |

**Additional File 1. Univariable analysis comparing 115 transplanted cirrhotics versus 106 cirrhotics listed but died waiting for transplant (two sites).**
