# Supplementary material for: Liver transplantation in the critically ill: a multicenter Canadian retrospective cohort study
Source: Crit Care. 2013 Feb 9;17(1):R28. doi: 10.1186/cc12508 (PMC4056692; doi:10.1186/cc12508)
Supplement: Additional file 2 — Multivariable analysis: Predictors of receipt of liver transplant in 221 critically ill cirrhosis patients on the transplant list in the ICU.Description: Multivariable (adjusted) predictors of liver transplantation (2 sites). [file cc12508-S2.DOCX]

| **Covariate** | **Unadjusted** | **Model 1(n=213)** | **Model 2(n=158)** | **Model 3 (n=126)** | **Model 4 (n=174)** | **Model 5 (n=125)** |
| --- | --- | --- | --- | --- | --- | --- |
| Age | 0.99(0.96-1.01) | 0.98 (0.96-1.01) | 0.98 (0.94-1.02) | **0.95 (0.91-0.99)*** | 0.98 (0.94-1.01) | **0.95 (0.91-0.99)*** |
| Gender (female) | 1.06 (0.59-1.89) | 1.21 (0.65-2.25) | 1.66(0.81-3.40) | 1.51 (0.64-3.57) | 1.02 (0.51-2.05) | 1.58 (0.66-3.80) |
| Etiology (HCV vs. non-HCV) | 1.28 (0.72-2.28) | 1.62(0.87-2.99) | 1.68 (0.83-3.43) | 1.78 (0.76-4.14) | 1.63 (0.81-3.28) | 1.72 (0.73-4.03) |
|  |  |  |  |  |  |  |
| SOFA (admission) | **0.94 (0.88-0.99)*** | 0.96(0.89-1.04) | 1.04 (0.94-1.15) |  |  |  |
| Lactate (admission, natural log) | **0.52 (0.34-0.79)*** |  | **0.49(0.30-0.78)*** |  |  |  |
|  |  |  |  |  |  |  |
| SOFA (48 hours) | **0.88 (0.82-0.94)*** |  |  |  | **0.89(0.82-0.97)*** | 0.92(0.82-1.04) |
| 48 hour Lactate (natural log) | **0.30 (0.17-0.53)*** |  |  | **0.29 (0.15-0.53)*** |  | **0.32 (0.17-0.61)*** |
|  |  |  |  |  |  |  |
| Χ^2^ squared (degrees of freedom) |  | 16.37 (6) | 16.65 (7) | 31.03 (6) | 23.75(6) | 33.23 (7) |

**Additional File 2. Multivariable Analysis: Predictors of receipt of liver transplant in 221 critically ill cirrhotics on transplant list in ICU**

- All 5 multivariable models were adjusted for site of transplant, decade of transplant (2000-2009 vs. 1990-1990), individual data not shown.
- Lactate (on admission and at 48 hours) was converted to a natural logarithm to meet assumptions of logistic regression (normal distribution)
- Hosmer Lemeshow goodness of fit p> 0.3 for all models.
- All models included age, gender (female), etiology (HCV vs. non-HCV), site (2 sites), decade of transplant
- Model 1 (n=213 of 221 patients) included SOFA score on admission
- Model 2 (n=158) included SOFA score and lactate (natural logarithm) on admission
- Model 3 (n=126) included natural logarithm of lactate at 48 hours
- Model 4 (n=174) included SOFA score 48 hours after admission
- Model 5 (n=125) included SOFA score and lactate (natural logarithm) 48 hours after admission
- **Significant results (p values):**
  - **Age: Model 3(p=0.036), Model 5(p=0.043)**
  - **SOFA (admission): Unadjusted (p=0.025)**
  - **SOFA (at 48 hours): Unadjusted (p<0.001), Model 4 (p=0.006)**
  - **Lactate admission (natural logarithm): Unadjusted (p=0.002), Model 2 (p=0.003**)
  - **Lactate 48 hours after admission (natural logarithm): Unadjusted (p<0.001), Model 3 (p<0.001), Model 5(p=0.001)**
